# The Radical Prostatectomy Versus Brachytherapy for the Management of Low- and Intermediate-Risk Prostate Cancer: A Meta-Analysis of Observational Studies Focusing on Oncological Outcomes

**DOI:** 10.31557/APJCP.2026.27.1.233

**Published:** 2026-01-22

**Authors:** Syah M. Warli, Muhammad F. Ikram, Dhirajaya D. Kadar, Ginanda P. Siregar, Fauriski F. Prapiska, Lidya I. Laksmi, Zaimah Z. Tala, Dewi IS. Siregar, Mutiara I. Sari Siregar

**Affiliations:** 1 *Department of Urology, Universitas Sumatera Utara Hospital, Jl. Dr. Mansyur, No. 5, Medan, Indonesia. *; 2 *General Practitioner, Latemmamala General Hospital, Soppeng, Indonesia.*; 3 *Department of Anatomy Pathology, Faculty of Medicine, Universitas Sumatera Utara, Medan, Indonesia.*; 4 *Department of Nutrition, Faculty of Medicine, Universitas Sumatera Utara, Medan, Indonesia.*; 5 *Department of Clinical Pathology, Faculty of Medicine, Universitas Sumatera Utara, Medan, Indonesia.*; 6 *Department of Biochemistry, Faculty of Medicine, Universitas Sumatera Utara, Medan, Indonesia.*

**Keywords:** Brachytherapy, low-intermediate risk, radical prostatectomy, prostate cancer, oncological outcome

## Abstract

**Objective:**

This study aims to compare the oncological outcomes of radical prostatectomy (RP) and brachytherapy (BT) in patients with low- and intermediate-risk prostate cancer, and determine whether one treatment demonstrates superiority over the other.

**Methods:**

A systematic literature search was conducted using databases, including PubMed, ScienceDirect, Google Scholar, EBSCO, and the Cochrane Library, to identify relevant clinical studies. Hazard ratios (HRs) and 95% confidence intervals (CIs) were extracted, with HRs >1 indicating RP superiority and HRs <1 indicating BT superiority. Outcomes assessed included biochemical relapse-free survival (BCRFS), clinical relapse-free survival (CRFS), overall survival (OS), and cancer-specific survival (CSS). Statistical analyses, including heterogeneity, publication bias, and risk of bias, were performed using R Studio 4.3.3 and Review Manager (RevMan) 5.4.

**Result:**

A total of seven studies involving 5663 patients were included in the analysis, with 2389 patients receiving brachytherapy (BT) and 3274 undergoing radical prostatectomy (RP). The pooled results demonstrated that BT was associated with significantly better biochemical relapse-free survival (BCRFS) compared to RP, with an HR of 0.84 (95% CI: 0.78–0.89; p<0.01). Although clinical relapse-free survival (CRFS) also favored BT, the result was not statistically significant (HR 0.90; 95% CI: 0.77–1.05; p=0.17). For overall survival (OS) and cancer-specific survival (CSS), the differences between the two treatment modalities were not statistically significant, with HRs of 1.08 (95% CI: 0.87–1.34; p=0.50) and 1.05 (95% CI: 0.82–1.36; p=0.70), respectively. Subgroup analyses based on risk stratification and follow-up duration revealed variability in treatment outcomes, particularly favoring BT in certain intermediate-risk groups.

**Conclusion:**

This meta-analysis suggests that brachytherapy may offer superior outcomes in biochemical and clinical relapse-free survival compared to radical prostatectomy in patients with low- and intermediate-risk prostate cancer. However, no significant differences were observed in overall survival or cancer-specific survival, highlighting the need for individualized treatment decision-making based on patient risk profiles and preferences.

## Introduction

Through the years, the discussion revolving around prostate cancer has almost invariably centered to its high mortality rate, hence prostate cancer has emerged as one of the most significant health challenges for men globally, both in terms of incidence and mortality [1]. Data from recent decades clearly indicate that prostate cancer is now one of the five most prevalent cancers on a global scale, ranking third according to the GLOBOCAN (Global Cancer Observatory) database [2]. Indeed, it is also the fifth leading cause of cancer related deaths, accounting for a massive 1.4 million new cases reported in 2020 onwards alone [3, 4]. This contributed to approximately 7.3% of all cancer cases worldwide, affecting approximately 375,000 deaths within that year [5]. A concerning aspect of prostate cancer is the steadily increasing prevalence and mortality rates over the years [6]. This tendency is mainly noticeable in developed countries, where the burden of the disease is growing year-on-year [7]. These escalating figures highlight the need for continued attention and systematic effort to address this health issue. The urgency of this situation warrants that prostate cancer becomes a primary subject of ongoing research and discourse, specializing in urology. In response to this growing health crisis, the American Urological Association (AUA) released its 2022 guidelines, outlining various therapeutic modalities for the management of prostate cancer [8]. These recommendations are particularly pertinent for patients categorized as low to intermediate risk, a classification determined by factors outlined by the Gleason score, clinical radiological assessments, and blood Prostate-Specific Antigen (PSA) levels [9]. Among the therapeutic options, two approaches stand out for their frequent consideration in clinical practice: radical prostatectomy (RP) and brachytherapy (BT), which involves the surgical removal of the entire prostate gland, has long been regarded as the gold standard treatment for many prostate cancer patients. This procedure is particularly valued for its ability to achieve definitive local control of the cancer [10-12].

By the same token, BT provides a treatment option which does not require operative procedures. This technique involves direct implantation of radioactive seeds into the prostate, allowing for localized radiation treatment [13-15]. However, despite its less invasive nature, the long-term efficacy of BT remains a subject of ongoing research and debate. There are concerns regarding its effectiveness in providing sustained cancer control, as well as its potential impact on the patient’s quality of life in the long run. The debate over which treatment approach RP or BT offers superior oncological outcomes is far from settled. This discussion is particularly important when considering key metrics such as overall survival rates, disease-free survival, and freedom from cancer recurrence [16]. The available observational studies have yielded mixed results, with some suggesting comparable outcomes between the two treatments, while others highlight differences in survival rates, recurrence, and side effects [17-19]. Given the rising burden of prostate cancer and the existence of multiple treatment modalities, evaluating the comparative effectiveness of these interventions becomes crucial.

Understanding the nuanced differences in oncological outcomes, including disease-free survival and life expectancy, is crucial for improving patient care. This meta-analysis seeks to delve deeper into the comparative effectiveness between RP compared to BT, particularly in patients with prostate cancer concerning to low and intermediate risk. The goal is to determine whether one treatment offers superior oncological outcomes over the other, thereby informing more personalized and evidence-based clinical decision making.

## Materials and Methods

### Protocol registration

This protocol is registered under PROSPERO as the International Prospective Register of Systematic Reviews, with the ID CRD42024584671.

### Study selection criteria

This meta-analysis leveraged the PICO strategy to assess the superiority of oncologic outcomes of RP compared to BT therapy as a treatment option for improving survival conditions and status in patients diagnosed with low- and intermediate-risk prostate cancer by The National Comprehensive Cancer Network (NCCN) Criteria. Therefore, the PICO strategy for this meta-analysis is as follows: (1) Population: patients diagnosed with low- and intermediate-risk prostate cancer, defined by PSA levels, histopathology biopsy (Gleason Score), and clinical staging, (2) Intervention: Radical Prostatectomy (RP), (3) Comparator: Brachytherapy (BT), and (4) Outcomes: oncological outcomes including Biochemical Relapse-Free Survival (BCRFS), Clinical Relapse-Free Survival (CRFS), Overall Survival (OS), and Cancer-Specific Survival (CSS). The focus is on observational studies, preferably those conducted on a small to large scale with explicit protocols. During the literature identification process, studies were excluded if they had incompatible trial designs (e.g patients had been treated first with one of the treatment or not had been done simultaneously, unsuitable outcome if the reported outcomes did not match our predefined oncological endpoints, if the comparator variables are not related to the purpose of the study or if they had incomplete data reporting.

### Database searching and systematic literature screening

All of the authors conducted a comprehensive literature search using medical electronic databases such as PubMed, ScienceDirect, Google Scholar, EBSCO, and the Cochrane Library, for study screening. This review was conducted from from January 2024 to August 2025. A Boolean approach was utilized to correlate the keywords in the title-based abstract and identification, i.e. “Radical Prostatectomy” OR “RP” AND “Brachytherapy” OR “BT” AND “Prostate Cancer” OR “PCa” AND “Oncological Outcome”. Each of the following keywords has some functional synonyms or abbreviations for example “BT” for the frame “Brachytherapy” and “RP” for the form “Radical Prostatectomy which is incorporated through the ‘OR’ keywords thereof. The detailed search strings, Boolean operators, and combined syntax used for the literature search are summarized in Table 1. The reference lists of articles are manually screened, and there are no meta-analysis studies pertinent to the goal of obtaining any conceivable literature. This review based on Preferred Reporting Items for Systematic Review and Meta-analysis (PRISMA) protocol. Eligibility criteria for this study included: observational cohort studies according to PICO, full-text articles, complete data reporting, written in English. Decade-plus old studies were included in this review. Engage in a process of finding and screening literature, the obtained studies were compiled into a database. Duplicate articles were removed, and the remaining articles underwent further screening based on the required format. Full-text articles were retrieved for the selected studies, and the eligibility of the articles was assessed based on their titles and abstracts. For articles that passed this initial selection stage, the entire manuscripts were read.

### Statistical design and analysis

Various approaches were employed to interpret and conduct our mathematical and structured analysis in this study, and the parameters were extracted from Hazard Ratio (HR) and Standard error (SE) values in each survival outcome measurement using Microsoft Excel software, the statistical software R Studio 4.3.3 (R.4.3.3) and Review Manager (Revman) 5.4. The specific comparator variable used in this review was BT, which consist of Low-Dose Rate Brachytherapy (LDR-BT), and Seed Brachytherapy (SEED-BT). The inverse variance methods are implemented in several studies to analyze HR and Standard Error (SE) values for most outcomes. The overall heterogeneity of the outcomes was assessed using the I^2^ value which formulated by the DerSimonian-Laird estimator in R studio. Heterogeneity was considered low if the I^2^ value was <30.0%, Intermediate or with some concern if it ranged between 30.0-50.0%, and high or substantial if it was >50.0%. Random-effects model (REM) and Fixed-effects model was used for several outcomes when the I^2^ value exceeded 50.0% and less than 50.0%, respectively. Jakson formula was used in R Studio to a determined the 95% Confidence interval (95%CI). The p value of < .05 was considered to be statistically significant for both outcomes and all sub-analysis conducted.

### Data extraction

This meta-analysis includes patients with low and intermediate risk prostate cancer, classified according to NCCN guidelines [32]. Stratification is based on clinical assessments, including PSA levels, Gleason scores from histopathological prostate biopsies, and clinical staging. Low-risk patients are identified by T1-T2a staging, a Gleason score of ≤6, and PSA levels below 10 ng/ml. Intermediate-risk patients are characterized by T2b-T2c staging, a Gleason score of 7, or PSA levels ranging from 10-20 ng/ml, with favorable intermediate-risk defined as having fewer than 50% positive biopsy cores.

The main data investigated in the meta-analysis primarily revolved around the oncological outcome specifically in patient’s survival outcomes including BCRFS, CRFS, OS, and CSS. BCRFS alludes to the duration for which a patient remains free of biochemical recurrence. CRFS is described either as being cancer-free as identified by medical imaging, with no locally occurring symptoms, or as a biopsy-proven local recurrence. OS is defined as the time span from the date of diagnosis to the occurrence of death or the last follow-up, irrespective of the cause of death. CSS is defined as death attributable to prostate cancer (Pca), as indicated on the death certificate, supported by biochemical and clinical data, or the presence of uncontrolled metastatic disease at the time of death. Outcomes in these studies are reported under the formula Hazard Ratio and Event= RP/BT, in which HR less than 1 means BT is favorable and greater than 1 means RP is favorable. 

### Sensitivity analysis

The latter analysis will facilitate determining potential resources of heterogeneity sources. This prompted further sensitivity analyses using subgroup methods, such as excluding potential sources of heterogeneity, to determine whether studies with significant differences could impact our final conclusions. This approach also enhances the robustness of the analysis by evaluating the confidence in the findings, effectively minimizing reporting bias and striving to make the data as homogeneous as possible. This, in turn, influences the certainty of the estimated outcomes in this study. Sensitivity analysis will be carried out by conducting several sub-analyses, including: 1) subgroup analyses based on follow-up durations (5, 8, and 10 years); and 2) analyses focused solely on intermediate-risk patients, ensuring that the choices treatment intervention was in intermediate risk according to AUA Guideline and the range of variation remains limited [8].

### Risk of bias and study quality

This systematic review and meta-analysis exclusively included observational cohort studies, with study quality assessed using the Newcastle Ottawa Scale (NOS). The NOS is a standardized tool designed to evaluate the methodological quality of non-randomized studies, particularly in systematic reviews. It assesses three key domains: Selection (four components), Comparability (one component), and Outcome (three components), with a total possible score of nine points. Criteria evaluated include the representativeness of the cohorts, the control of confounding variables, the method of outcome assessment, and the adequacy and duration of follow-up. Each study’s NOS score was then interpreted using The Agency for Healthcare Research and Quality (AHRQ) guidelines, which categorize studies as good, fair, or poor quality. This structured assessment ensures consistency and transparency in evaluating the risk of bias and the overall validity of the included studies. 

### Publication bias

The publication bias was assessed with the funnel plot and the asymmetry of the plot was analyzed by Egger’s test in R studio software for HR. p value <.05 was considered to be significant bias with the asymmetrical form of the funnel plot and >.05 for no significant bias founded. 

### Meta-Regression

Meta-regression was employed in the present investigation as an advanced analytical strategy to delineate the extent to which patient-level characteristics modulate key clinical outcomes following radical prostatectomy and brachytherapy in individuals diagnosed with prostate cancer. The four primary endpoints assessed comprised biochemical relapse-free survival (BCRFS), clinical relapse-free survival (CRFS), cancer-specific survival (CSS), and overall survival (OS). A mixed-effects meta-regression model, utilizing the restricted maximum likelihood (REML) method for estimating tau², was applied to accommodate both fixed effects of moderators and random effects accounting for between-study variability. The moderators under scrutiny included mean patient age, initial serum PSA concentration, intermediate-risk classification, and geographical region (non-Asian versus Asian). The consistent finding of a tau² value equal to zero across all models denotes the complete absorption of inter-study heterogeneity by the incorporated covariates, attesting to the statistical robustness and adequacy of the specified models. 

## Results

### Literature search

**Figure 1:**
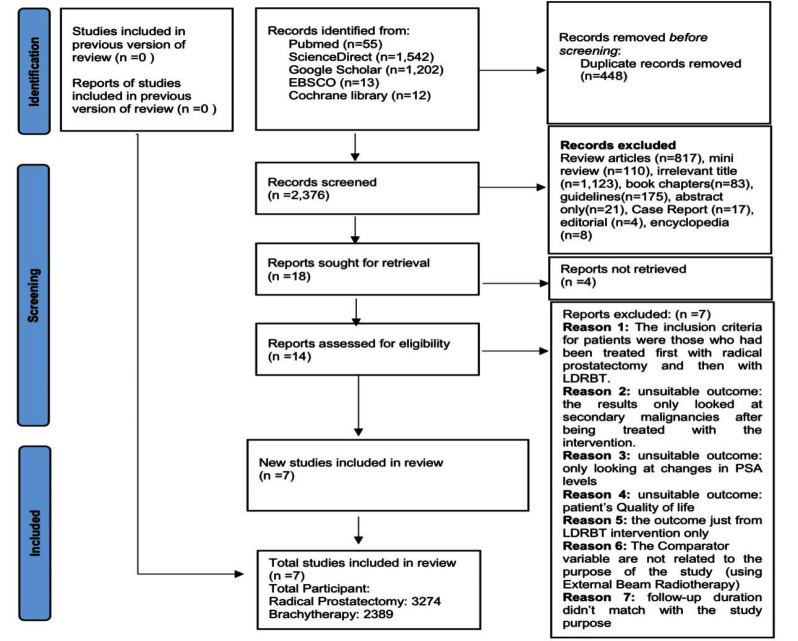
PRISMA 2020 Flow Diagram Used to Identify the Analyzed Study in This Review

According to the standard PRISMA protocol as the foundation of this study, the initial search yielded 2,824 articles. After removing 448 duplicated articles, 2,376 articles were left for title and abstract screening. Out of these 2,376 articles, 2,358 did not meet the required form of the article and were subsequently excluded. Consequently, the remaining 18 articles were sought for retrieval. Among them, the full text of 14 studies was accessible for further analysis. Out of the total of 14 studies initially identified, seven studies were excluded from the systematic review due to various reasons. These reasons included patients were those who had been treated first with RP and then with LD-RBT, the comparator variable not being related to the purpose of the study and having incomplete follow-up period. This is presented in Figure 1.

Following the last screening method, seven studies met the inclusion criteria [15,20-25]. All of these studies were cohort studies, aligning with the PICO criteria, and were published within the last 15 years. Moreover, all of the included studies were available in full-text format. No additional inclusion studies were obtained from the previous review studies. Overall, the total sample size included in this review consisted of 5,663 patients. Finally, a manual screening was performed on the article and non-finding reference lists of previous systematic reviews and meta-analysis studies that were related to the intention to obtain any literatures that were plausible and included them as “studies from other sources or review sources.” The study and patient characteristics are summarized in Table 2 and Table 3. 

### Risk of bias from included studies

The quality assessment or risk of bias evaluation was conducted by an author (MFI), and the results are presented in Table 4. All of the included studies were observational cohort studies. To assess the risk of bias and quality assessment in each study, the NOS assessment tools specifically designed to cohort study were utilized. Among the included studies, two studies exhibited a fair quality of due to inadequate selection of how the questionnaires were measured and unclear reporting of trial outcomes. Another study also demonstrated suboptimal reporting of outcomes. In the remaining studies, there was a lack of clarity regarding the measurement of questionnaires. Disagreements in assessments were resolved through consensus or adjudicated by a third reviewer.

To evaluate the robustness of the findings, a sensitivity analysis was performed by excluding studies rated as “fair” or “poor” quality. Results remained consistent, indicating that lower-quality studies did not significantly influence the overall conclusions.

### Comparative Oncological Outcomes Following Radical Prostatectomy and Brachytherapy in Patients with Low- and Intermediate-Risk Prostate Cancer

**Figure 2:**
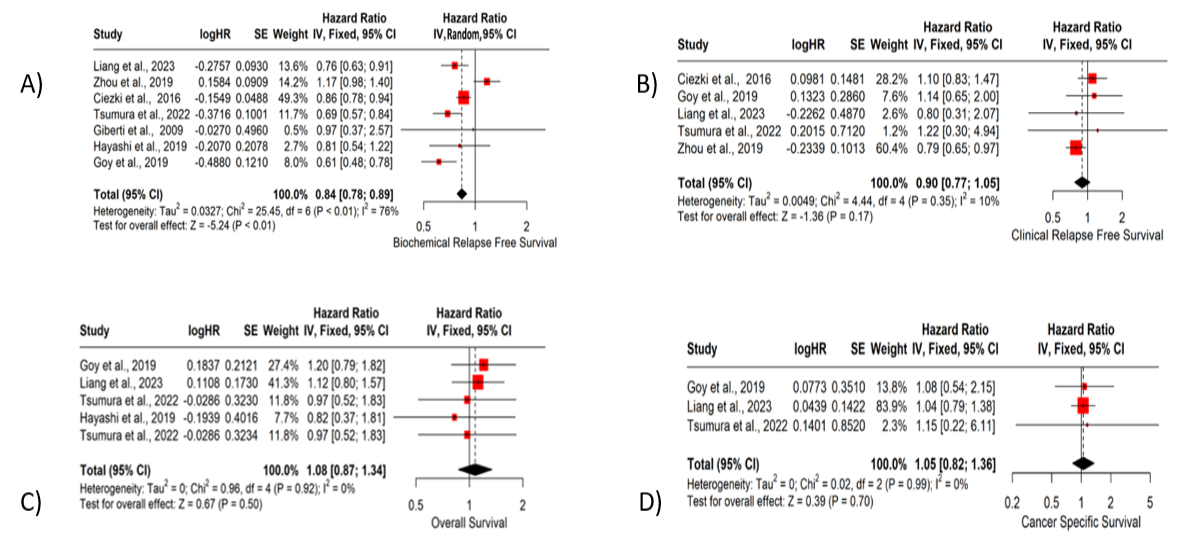
Meta-Analysis of (A) Biochemical Relapse-Free Survival (BCRFS); (B) Clinical Relapse-Free Survival (CRFS); (C) Overall Survival (OS); and (D) Cancer-Specific Survival (CSS) between Radical Prostatectomy and Brachytherapyin low- and intermediate-risk prostate cancer patient.

Based on the analysis involving seven studies, including 5,663 both low as follow as in all categories (low- and-intermediate risk) prostate cancer patients which 2,389 prostate cancer patients intervened by BT and 3,274 treated by RP showed that BT had significantly improved BCRFS as superior to RP (Pooled HR: 0.84; 95%CI: 0.78-0.89; p<0.01). Heterogeneity was found (p<0.01; I^2^ 76%) so the random effect model was used and there is no funnel plot asymmetry, which indicates no publication bias (p= 0.7195). The CRFS also revealed the superiority of the BT over the RP (Pooled HR: 0.90; 95%CI: 0.77-1.05; p=0.17) with low heterogeneity (p=0.35; I^2^=10%), hence a fixed effect model was used with no funnel plot asymmetry which indicated no publication bias (p=0.4589). However, contrasting results were demonstrated in the oncologic outcomes of OS and CSS which showed a superiority of RP over BT (HR: 1.08; 95%CI: 0.87-1.34; p=0.50 and HR: 1.05; 95%CI: 0.82-1.36; p=0.70) with no finding of heterogeneity (p=0.92; I^2^=0% and 0.35; I^2^=0%) (Figure 2), respectively, so fixed effect models were used. There was no funnel plot asymmetry which indicates no publication bias in OS and CSS (p=0.0518 and 0.0532, respectively) (Figure 2 and Table 5).

### Subgroup analysis

**Figure 3:**
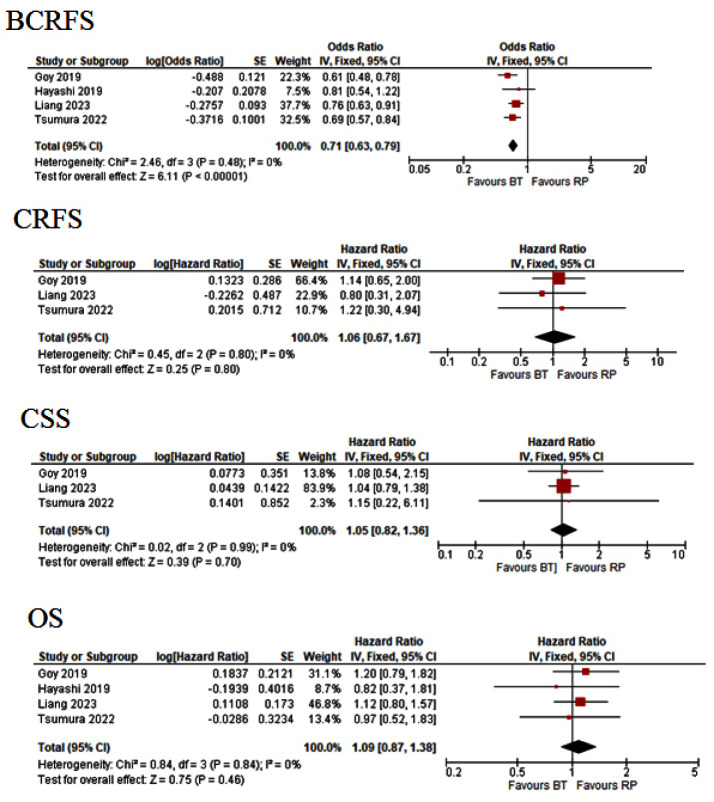
Meta-Analysis of (A) Biochemical Relapse-Free Survival (BCRFS); (B) Clinical Relapse-Free Survival (CRFS); (C) Overall Survival (OS); and (D) Cancer-Specific Survival (CSS) between Radical Prostatectomy and Brachytherapy in intermediate-risk prostate cancer patient.

Subgroup analysis was carried out to reveal the oncological outcome only in intermediate risk patients. Based on the analysis, the hazard ratio in BCRFS, CRFS, CSS, and OS between BT versus RP was 0.71(95%CI: 0.66-0.86; p<0.001), 1.06(95%CI: 0.85-1.39; p=0.8), 1.05(95%CI: 0.82-1.36; p=0.7), 1.09(95%CI: 0.87-1.38; p=0.46), respectively (Figure 3).

In intermediate-risk prostate cancer patients, BT significantly improved BCRFS compared to RP, indicating a clinically meaningful 29% reduction in biochemical recurrence. However, no significant differences were observed for CRFS, CSS, or OS, suggesting that long-term outcomes are comparable. These results support BT as a valid alternative to RP, particularly when prioritizing local disease control.

**Figure 4:**
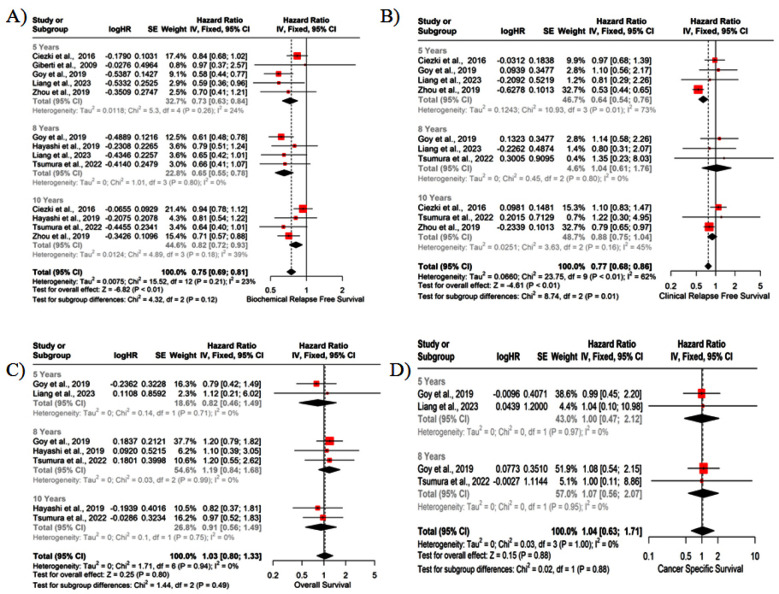
Subgroup Meta-Analysis of (A) Biochemical Relapse-Free Survival (BCRFS); (B) Clinical Relapse-Free Survival (CRFS); (C) Overall Survival (OS); and (D) Cancer-Specific Survival (CSS) in 5,8 and 10 years follow-up duration

Subgroup analysis based on duration of follow-up was performed for BCRFS, CRFS, OS, and CSS, comparing RP with LDR-BT. For BCRFS, the HRs at 5, 8, and 10 years were 0.73 (95%CI: 0.63-0.84), 0.65 (95%CI: 0.55-0.78), and 0.82 (95%CI: 0.72-0.93), respectively, with an overall HR of 0.75 (95%CI: 0.69-0.81; p<0.01), indicating the lower incidence of BCRFS in the RP group made it clear that BT was superior over RP. Heterogeneity was low (p=0.21; I²=23%), and no significant differences were found throughout follow-up duration (p=0.12). For CRFS, hazard ratios were 0.64 (95%CI: 0.54-0.76), 1.04 (95%CI: 0.61-1.76), and 0.88 (95%CI: 0.75-1.04), respectively, yielding an overall HR of 0.77 (95%CI: 0.68-0.86; P<0.01), indicating fewer CRFS events in the RP group, suggested that BT was also superior over RP. Significant heterogeneity was present (P<0.01; I²=62%), and there was a significant difference between follow-up durations (P=0.01). For OS, hazard ratios were 0.82 (95%CI: 0.46-1.49), 1.19 (95%CI: 0.84-1.68), and 0.91 (95%CI: 0.56-1.49), respectively, with a pooled HR of 1.03 (95%CI: 0.80-1.33; P=0.80), showing no significant difference in overall survival between groups. There was no heterogeneity (P=0.94; I²=0%), and follow-up duration did not significantly affect the outcome (P=0.49). For CSS, HRs at 5 and 8 years were 1.00 (95%CI: 0.47-2.12) and 1.04 (95%CI: 0.56-2.07), with a pooled HR of 1.04 (95%CI: 0.63-1.71; P=0.88), and there was no significant difference in CSS between groups. No heterogeneity was detected (P=1.00; I²=0%), and no significant differences were observed across follow-up durations (P=0.88) (Figure 4).

Subgroup meta-analysis was conducted to assess long-term outcomes of LDR-BT versus RP across 5, 8, and 10 years of follow-up. For BCRFS, the pooled HR was indicating a 25% lower risk of biochemical recurrence with BT. Clinically, this is significant, suggesting BT provides better biochemical disease control over time. For CRFS, the pooled HR was 0.77, favoring BT. Although there was heterogeneity across time points, this suggests a 23% lower risk of clinical relapse, which is also clinically meaningful.

In contrast, for OS and CSS, the pooled HRs were respectively both statistically and clinically non-significant, indicating no survival advantage for either treatment. These findings imply that while BT may improve recurrence outcomes, it does not appear to impact long-term survival compared to RP.

### Meta-regression Analysis

The meta-regression analysis assessing factors influencing BCRFS included data from seven studies. The overall pooled HR for BCRFS was 0.84 (95% Confidence Interval: 0.78–0.89), indicating a statistically significant reduction in the risk of biochemical relapse (p < 0.01). However, there was notable heterogeneity among the studies, with an I² value of 76%, prompting further exploration through meta-regression (Supplementary Table 1).

Several covariates were analyzed for their potential impact on BCRFS. The initial mean PSA level had a positive coefficient estimate (0.0656), but this association was not statistically significant (p = 0.2293), suggesting it may not be a strong independent predictor of BCRFS. Similarly, the region (non-Asian vs. Asian studies) showed no significant effect (coefficient = 0.8404, SE = 1.3186), and mean age also did not demonstrate a significant association (coefficient = 0.2061, p = 0.4604). Interestingly, the intermediate-risk group showed a negative coefficient (-0.6103), with a p-value of 0.0580, indicating a potential trend toward worse BCRFS outcomes in this subgroup, although it did not reach conventional statistical significance. Overall, while the model identified some trends, no covariate except the overall HR reached clear statistical significance in explaining the observed heterogeneity.

In the meta-regression analysis of CRFS (Supplementary Table 2), based on seven studies, the pooled hazard ratio was 0.90 (95% CI: 0.77–1.05), which was not statistically significant (p = 0.17), and the heterogeneity was low (I² = 10%). Among the examined covariates, initial mean PSA showed a significant positive association with CRFS (coefficient = 0.3040, p = 0.0004), indicating that higher PSA levels at baseline were associated with an increased risk of clinical relapse. The intermediate-risk group also demonstrated a statistically significant negative coefficient (-0.7722, p = 0.0390), suggesting worse CRFS outcomes in this subgroup. However, region and mean age did not show significant associations (p = 0.2273 and 0.1528, respectively).

For OS (Supplementary Table 3), the meta-regression included the same number of studies and showed a pooled HR of 1.08 (95% CI: 0.87–1.34), with low heterogeneity (I² = 10%) and no significant overall effect (p = 0.5). Nonetheless, several covariates were significantly associated with OS. Interestingly, initial mean PSA had a negative coefficient (-0.2184, p = 0.0042), indicating better OS with lower baseline PSA values. The region (non-Asia) variable had a significant positive association (coefficient = 1.4167, p = 0.0427), suggesting poorer OS outcomes in studies conducted outside Asia. Both mean age (coefficient = 0.3740, p = 0.0441) and intermediate risk (coefficient = -0.9702, p = 0.0004) were also significant, with the former associated with worse survival and the latter indicating reduced OS in intermediate-risk patients.

In contrast, the meta-regression analysis of CSS (Supplementary Table 4) revealed no statistically significant associations across all covariates. The pooled HR was again 1.08 (95% CI: 0.87–1.34), with a p-value of 0.5 and low heterogeneity (I² = 10%). Neither initial mean PSA (p = 0.8043), region (p = 0.7645), mean age (p = 0.8334), nor intermediate risk (p = 0.6779) showed meaningful influence on CSS outcomes in this analysis, suggesting that the studied covariates did not substantially impact cancer-specific survival in the included studies.

## Discussion

This meta-analysis compared the oncologic outcomes of RP and BT in patients with low- and intermediate-risk prostate cancer. The findings indicate that BT showed better BCRFS than RP, particularly in patients with favorable prognostic factors such as lower Gleason scores and PSA levels. Conversely, RP appeared slightly superior in OS and CSS, though differences were often statistically non-significant. CRFS results varied, with no consistent advantage between the treatments. Subgroup analyses revealed that follow-up duration and patient characteristics significantly influenced outcome heterogeneity.

Since its inception by the AUA as a therapeutic modality for treatment of prostate cancer, the preference between RP and BT for the management of low- and intermediate-risk prostate cancer continues to be a pivotal and much-debated topic within the urology field [16, 26]. Historically, RP has been perceived as the gold standard and has proven effective in treating prostate cancer with high survival rates among low to intermediate risk patients [27]. Long-term studies have also demonstrated that RP boasts good cancer control and low recurrence rates, predominantly in patients with lower Gleason scores and preoperative PSA, until BT emerged as one of the other therapeutic modalities that is equally predicted to be as effective as RP [28, 29]. There are some studies showing that BT gives oncologic control comparable to RP in the short and medium term for patients with low to intermediate risk. Yet, there are variations in outcomes on the basis of the technique used and the experience of the surgeon [30]. BT is also advantageous due to the lack of invasive procedures, rapid recovery time, and comparatively few side effects compared to RP [17-19, 31]. 

The NCCN states that patients with prostate cancer can be classified and stratified by their risk level, utilizing assessment parameters which include blood PSA levels, Gleason score from histopathologic prostate biopsy examination and clinical stage, defined as low risk: T1-T2a, Gleason score ≤6, and PSA <10 ng ml-1; intermediate risk: T2b-T2c or Gleason score 7 or PSA 10-20 ng ml and <50% positive biopsy cores for favorable intermediate risk [32]. The effectiveness of both therapy approaches has been shown in various studies, most notably in oncologic control outcomes [17, 33, 34]. In the terminology of oncological control, it can also be classified as BCRFS, CRFS, OS, and CSS which between these two interventions have different superiority in each of their oncological outcomes, and again this meta-analysis study focuses on these two interventions for each of the oncological outcomes that have been described previously [18].

The first oncological outcome to be discussed is BCRFS, where an in-depth understanding of BCRFS can be more easily comprehended if the definition of biochemical failure (bF) is recognized. Referring to the AUA bF is the time interval from initial therapy to the occurrence of a rise in PSA levels above a certain threshold value, which indicates biochemical recurrence with a PSA value of ≥0.2 ng/ml on two consecutive measurements after the PSA fell to <0.2 ng/mL for patients who underwent RP and utilize the Phoenix definition as an increase of 2 ng/ml or greater nadir PSA value for patients receiving LDR so that the understanding of BCRFS is understandable as a patient free from biochemical failures [35] Several factors can contribute to BCRFS ranging from patient demographic characteristics, type and quality of therapy to the role of adjuvant therapy [36].

BCRFS is additionally used as a major indicator in evaluating therapeutic outcomes and patient prognosis. However, those with longer or bigger BCRFS are more likely to have better long-term outcomes and BCRFS can help determine whether patients require further interventions with hormonal therapy or chemotherapy [37]. The drawbacks of BCRFS, however, are that the lack of a universal standard for measuring and reporting BCRFS may impede comparisons between studies and it should be noted that an increase in PSA does not necessarily indicate clinical recurrence, and a stable PSA does not necessarily mean that the disease has been completely eliminated, so the concept of false positives and negatives still applies [38]. The results of a meta-analysis conducted on 7 studies focused on oncologic outcomes of BCRFS in low-risk prostate cancer patients intervened with BT showed favorable results in BCRFS compared to RP with a Pooled HR (0.84), suggesting BT is superior to RP. The researchers further “unraveled” some of the variables that may have influenced the previous pooled results by performing a subgroup analysis that demonstrated whether there was a difference in the pooled HR at each follow-up duration. At 5, 8, and 10 years of follow-up, the oncologic outcomes of BCRFS in low-risk prostate cancer patients intervened with BT remained better than those of RP with HR (0.73), (0.65), and (0.82), respectively. Interestingly, in addition to the HRs that may not be different from those without subgroups, the reported heterogeneity was much lower than those without subgroups, assuming the influence of the number of patients in each study or a patient population with more aggressive tumor characteristics [15, 20-25, 39]. 

The superior BCRFS observed with BT may be attributed to its ability to deliver a higher biologically effective dose directly to the prostate tissue through continuous, localized radiation. This allows for more precise tumor control, particularly in cases where the cancer is confined within the gland. In contrast, RP involves the surgical removal of the prostate, which can leave behind microscopic disease in surrounding tissues, potentially increasing the risk of biochemical recurrence [18, 20, 22]. Our findings are consistent with previous studies such as those by Ciezki et al. and Goy et al., which demonstrated improved BCRFS with BT. Additionally, studies by Zou et al. and Liang et al. further supported BT’s efficacy, particularly in patients with favorable prognostic features, such as lower PSA levels and Gleason scores. The study by Ciezki et al has the most dominating weight compared to other studies, as seen from the study where Ciezki et al also have reported positive results with this meta-analysis study by favoring BT compared to RP in intermediate- and low-risk patients for BCRFS outcomes but the Ciezki et al study has a very large total of patients and uneven distribution of patients, in prostate cancer patients intervened with BT only 515 people while RP more than twice with 1,308 people [20]. Perhaps this disparity in patient distribution makes this study one of the causes of high heterogeneity in pooled results with dominating weights. In the Goy et al study, BT also provided superior freedom from biochemical failure compared to RP at 5 years probability, which was 90.7% vs 73.0% and 10 years probability of 82.0% vs 58.0% [15]. An alternative approach taken by the authors in addition to exclude the Ciezki study so that the distribution of weights in the meta is equitable is to create a subgroup that is only devoted to cancer prostate patients with intermediate risk only, where the study obtained a pooled HR is 0.71 where BT still has superior results compared to RP but with lower heterogeneity [15,20-25]. Regarding patient characteristics in influencing BCRFS outcome, as in the study of Zou et al, BCRFS rates in LDR were significantly increased over RP among those patients with biopsy Gleason score ≤3+4 or iPSA ≤10 ng/ml. Considering the outcome of this research, it may be a better option for BT than for RP in patients with biopsy Gleason score ≤3+4 or iPSA ≤10 ng/ml. Regarding pretreatment attributes, patients treated with LDR tended to be older, experienced longer follow-up times, and had more combinations of androgen deprivation therapy [25]. This is also corroborated by a study Liang. et al that has observed BT remains superior to RP in BCRFS oncologic outcomes especially in patients with Gleason Score 7, PV>30 mL, initial PSA <10ng/mL and clinical staging T1c-T2a, regardless of the percentage positive biopsy cores (PPBC) value. Again, positive results showing the superiority of BT in BCRFS were revealed through a study in 5 years of follow-up duration compared to RP but the studies by Ferreira et al. and Taussky et al. reported in the form of Odds Ratio and percentage, although the study by Taussky et al. in 575 low- and intermediate-risk prostate cancer patients reported that BT and RP were still equally comparable to be tested for superiority [40, 41].

The notion of CRFS can also be referenced as distant metastasis-free survival where it is understandably defined as patients being free of metastases identified by medical imaging, with or without localized symptoms, or as biopsy-proven local recurrence [39]. In CRFS oncologic outcomes, the pooled meta-analysis also showed superiority of BT over RP with HR (0.90) with low heterogeneity in patients with low- and intermediate-risk prostate cancer. However, the reported pooled meta-analysis results were not significant and it is assumed that the difference in the HR of each reported study was small and there were conflicting variations in HR results between studies. CRFS, which tracks radiological or symptomatic recurrence, may not always correlate with biochemical indicators. The observed variability in CRFS outcomes might be due to differences in follow-up durations and patient risk profiles across studies [20, 24, 37]. For instance, in the study of Tsumura et al. in 428 patients intervened with both treatment options, RP showed superior outcome of CRFS compared to BT with HR (1.22), supported by the study of Ciezki et al. in 1,308 patients in the analysis. On the contrary, a study reported by Zhou et al. in 1,308 patients in a multivariate analysis of CRFS “apart” from the pooled results of meta-analysis in this study showed RP was significantly superior to BT therapy in low- and intermediate-risk prostate cancer patients with an HR of (1.57) [20, 24]. Conversely, the study reported by Zhou et al showed BT still provided superior results in terms of CRFS compared to RP [37]. The subgroups constructed to assess the superiority between the two interventions reported by duration of follow-up were peculiar in that at 5 and 10 years of follow-up, BT had significantly superior results in CRFS with HRs of 0.64 and 0.88, whereas at 8 years of follow-up RP had significantly superior results with a HR of 1.04 and it is notable that the differences between the subgroups were significant [15, 24, 39]. Some studies were also removed and categorized them into subgroups which only considered CRFS in intermediate risk prostate cancer patients. The four studies analyzed showed RP to be superior to BT with an HR 1.06. Nevertheless, it is important to focus on the fact that heterogeneity in this subgroup reached 0%, which is not a desirable result in an analysis where it may be due to the lack of distribution in the patient population or the study methods and protocols that were reported [15, 20, 24, 39].

If focused on oncologic outcomes OS, it is defined as the time from the date of diagnosis to death or last follow-up, without limitation of the cause of death [41]. The pooled meta-analysis showed the superiority of RP over BT with HR 1.80 with low heterogeneity in low- and intermediate-risk prostate cancer patients as the reported pooled meta-analysis results were not significant. The lack of significance in the pooled meta-analysis results may be related to the variability of results in OS oncology outcomes where two out of five studies with HR 1.20 and 1.12 showed RP to be superior while the other three studies showed the opposing results with HR 0.97, 0.82, and 0.97 regardless of the non-significance of the results. The superiority of RP was also revealed in the OS subgroup analysis which was conducted based on the duration of follow-up with an HR of 1.03. There were several duration of follow-up which showed contrasting results at 5 and 10 years with HRs of 0.82 and 0.91, respectively. Comparable pooled results were also revealed in another subgroup that included only intermediate risk patients with HR of 1.09. However, the results obtained in this subgroup were not significant. This advantage may reflect a selection bias, wherein healthier, younger patients are more often directed toward surgery, while BT is typically offered to older or more comorbid individuals [25, 42]. These differences in baseline health status can influence non-cancer mortality and thus impact overall survival statistics. Earlier reports, such as those by Zhou et al., noted improved OS with RP, potentially due to fewer non-cancer-related deaths. Nonetheless, this advantage in OS should be interpreted cautiously, as it may not directly reflect treatment efficacy for prostate cancer itself [25].

Goy et al. was also reported on the same issue as the findings of this meta-analysis which demonstrated no significant difference between OS and CSS between the two treatment groups, possibly due to excess deaths unrelated to prostate cancer, and perhaps owing to the higher utilization of salvage therapy in RP. Several factors why RP was superior in OS found in several studies were also likely due to younger age in patient characteristics and deaths unrelated to prostate cancer. Some of the causes of death unrelated to prostate cancer in BT treatment were heart disease and cerebral hemorrhage while in RP many patients died from other cancers such as laryngeal and gastric cancer which may be complications of prostate cancer or adverse effects of the therapy [15, 22].

The cause of mortality in prostate cancer, either as confirmed on the mortality certificate along with biochemical and clinical related information, or the uncontrolled existence of metastatic disease at the onset of death, which is the conceptual understanding of CSS [42, 43]. Similar to OS, RP is still the “winner” in CSS with an HR of 1.05. Within this series, one further contributing variable that might illuminate the discrepancy in prostate cancer-specific mortality (PCSM) is that our EBRT patients had a longer median follow-up time than either LDR or RP patients. The observed rate of PCSM may shed some lights on future directions for high-risk prostate cancer clinical research [44]. Specifically, the issue of intensification of local vs. adjunctive systemic therapy becomes pertinent. In the study of Goy et al. disclosed contrasting results in which BT had proportionately more T2b patients, having the most significant effect on the hazard ratio in prostate cancer-specific survival (PCSS) [15]. In a recent study, the team from Hamdy and his colleagues presented their 10-year survival results for localized prostate cancer which were managed mainly through active surveillance, surgery, or even radiotherapy, as it demonstrated no significant difference in CRFS, CSS, or OS between the approach of surgery and radiotherapy. Nonetheless, as this research included patients from all risk categories, it is unlikely to draw specific conclusions about IRPC diagnosed with a panel of both invasive and non-invasive examinations such as the new findings leveraging canine olfactory sensory utilization in detecting patients with prostate cancer regardless of its stratification [45, 46]. From a research standpoint, it is still strongly recommended to establish more long-term studies and randomized clinical trials or observational studies to better comprehend the advantages and limitations of each therapeutic modality. A well-designed prospective study that considers the same patient characteristics and demographics may provide more robust results on oncologic outcomes as well as other variables such as quality of life, and side effects of RP versus BT. This concept is thus expected to continue to improve clinical practice based on stronger scientific evidence. In enhancement, considerations about the cost and accessibility of therapy also influence the choice between RP and BT. RP, especially with the use of robotic technology, can be a more expensive option compared to BT. Yet, the potential additional costs associated with long-term care and side effect management should be considered in the context of overall cost-effectiveness. From a health policy stance, a clear understanding of the clinical and economic outcomes of these two modalities is essential for informed decision-making. All in all, the choice between the treatments should be based on a thorough discussion between clinicians and patients. Considerations include not only oncologic outcomes and risk of complications, but also patient preferences and the specific clinical situation. A comprehensive, evidence-based approach can help patients achieve the optimal balance between cancer control and quality of life, ensuring that they receive the best treatment according to their individual needs.

Taken together, the clinical implications of this meta-analysis highlight brachytherapy (BT) as a compelling treatment option for patients with low- and intermediate-risk prostate cancer. The hazard ratio (HR) of 0.84 for biochemical relapse-free survival (BCRFS) indicates a statistically significant 16% relative reduction in biochemical recurrence with BT compared to radical prostatectomy (RP). While this effect size may seem modest, it is potentially clinically meaningful, particularly for patients who prioritize avoiding further interventions such as salvage radiation or hormonal therapy. For many, delaying recurrence even by a few years can significantly impact quality of life and reduce treatment-related morbidity. The HR of 0.90 for clinical relapse-free survival (CRFS) suggests a 10% lower risk of clinical progression with BT, though this result was not statistically significant; still, it may carry clinical weight depending on patient age and comorbidity burden. Importantly, overall survival (OS) and cancer-specific survival (CSS) showed no significant difference between BT and RP (HRs of 1.08 and 1.05, respectively), reinforcing the interpretation that both treatments offer comparable long-term mortality outcomes. These findings suggest that treatment decisions should be tailored not only to oncologic risk but also to patient preferences, quality-of-life goals, and tolerance for potential side effects. In this context, BT presents itself as a less invasive yet oncologically sound alternative, especially attractive for patients who prioritize functional preservation and lower procedural risk.

With regard to the BCRFS outcome, the meta-regression model yielded a statistically significant omnibus moderator test (QM = 24.93; p = 0.0001); however, none of the individual covariates attained conventional statistical significance. Of particular note, the intermediate-risk category approached the threshold of significance (β = –0.6103; p = 0.058), suggesting a potential trend toward diminished biochemical control in this subset of patients. Clinically, this is highly consequential within urologic oncology, as the intermediate-risk group frequently represents a gray zone in therapeutic decision-making. These findings imply the necessity for intensified post-treatment surveillance or the consideration of adjuvant modalities in this risk stratum. The apparent lack of association between baseline PSA or age and BCRFS may reflect the predominance of pathologic and surgical variables such as margin status or extracapsular extension in determining biochemical recurrence rather than demographic indices per se. [15, 20-22]

In contrast to BCRFS, the analysis of CRFS identified two statistically significant predictors: initial PSA level (β = 0.3040; p = 0.0004) and intermediate-risk status (β = –0.7722; p = 0.0390). These findings carry considerable weight in the urological domain, as clinical recurrence post-definitive therapy is often indicative of microscopic metastatic dissemination or incomplete local control. Elevated pre-treatment PSA, a surrogate for tumor burden, plausibly portends inferior clinical remission. From a therapeutic perspective, this reinforces the need for a tailored multimodal strategy potentially incorporating salvage radiation or systemic androgen deprivation in patients presenting with high PSA values at diagnosis [22]. The deleterious association observed for the intermediate-risk cohort further highlights the inadequacy of current risk stratification schemes and underscores the potential value of substratifying this group based on additional parameters such as Gleason pattern or tumor volume [25].

Regarding the OS endpoint, nearly all evaluated moderators exhibited statistically robust associations with survival, accentuating their prognostic salience. Mean age demonstrated a counterintuitive positive association with OS (β = 0.3740; p = 0.0441), a phenomenon that might be attributable to selection bias wherein only physiologically robust elderly patients are included in curative trials. The inverse relationship between baseline PSA and OS (β = –0.2184; p = 0.0042) corroborates the oncological axiom that higher PSA denotes more aggressive or advanced disease. Interestingly, residence in non-Asian regions conferred a survival advantage (β = 1.4167; p = 0.0427), which could reflect discrepancies in healthcare infrastructure, accessibility to high-quality oncology services, or differential adherence to clinical guidelines. Notably, intermediate-risk classification exerted a highly significant negative effect on OS (β = –0.9702; p = 0.0004), further affirming the vulnerability of this group and supporting the need for re-examination of therapeutic intensity for these patients [15].

In contradistinction, the CSS model failed to yield any statistically significant associations, either on the aggregate (QM = 2.73; p = 0.7420) or individual moderator level. The null findings in this context might be attributable to multiple causes, including limited statistical power due to a modest number of contributing studies (n = 7), a narrow range of CSS outcomes across studies, or the relative insensitivity of demographic variables to this endpoint. From a urological standpoint, CSS is intrinsically linked to oncological control and is more plausibly influenced by histopathological factors such as seminal vesicle invasion, extracapsular extension, or margin positivity. These findings underscore the limitations of relying solely on demographic or clinical surrogates and suggest the need for incorporating molecular markers or imaging biomarkers to refine CSS prognostication [15, 22].

From a statistical perspective, the capacity of each model to explain 100% of observed heterogeneity (R² = 1.0) is noteworthy and indicates that the specified moderators are not merely ancillary but rather central to the inter-study variance structure. Nevertheless, elevated standard errors observed in certain covariates most notably the geographical region in BCRFS and OS models necessitate cautious interpretation, given the possibility of wide confidence intervals and reduced inferential precision [22, 25]. The potential for multicollinearity between moderators also warrants consideration, as does the heterogeneity in outcome definitions across primary studies. Notwithstanding these caveats, favorable deviance values and parsimonious AIC metrics reinforce the models’ goodness-of-fit and underscore their empirical adequacy [15, 21-25].

The clinical ramifications of these findings are manifold. The consistent predictive utility of initial PSA in relation to both CRFS and OS affirms its central role in contemporary prostate cancer risk stratification systems, including those endorsed by the NCCN and EAU. Conversely, the recurrently detrimental impact of intermediate-risk status across most outcomes mandates a recalibration of therapeutic paradigms [25, 41, 42]. This group may benefit from intensified imaging (e.g., PSMA PET/CT), the integration of genomic risk scores, or the early initiation of adjuvant interventions. These findings collectively advocate for a more individualized treatment schema rather than a one-size-fits-all approach, particularly in a disease characterized by substantial biological heterogeneity.

In summation, this meta-regression elucidates the nuanced interplay between baseline patient characteristics and therapeutic outcomes following radical interventions for prostate cancer. The results not only provide clarity regarding which variables are most predictive of survival and disease control, but also carry profound implications for the personalization of oncologic care. Importantly, these insights reinforce the imperative for granular reporting in primary studies particularly regarding demographic and clinical covariates to facilitate future meta-analytic refinement. The analytical approach employed herein illustrates the critical role of advanced statistical methodologies in bridging the gap between aggregated evidence and individualized clinical decision-making in urologic oncology.

These results have important clinical implications. For patients with low-risk prostate cancer, BT should be considered a front-line treatment, particularly for those seeking less invasive therapy with lower risk of biochemical recurrence. For intermediate-risk patients, a personalized approach is essential. Those with favorable features may do well with BT, while others may benefit more from RP or combination therapy. Shared decision-making should play a central role, integrating patient preferences, comorbidities, and long-term quality-of-life goals.

There are several limitations to this meta-analysis. First, PI (I^2^) values were not reported in this analysis. This decision was primarily due to the limited number of included studies (n = 7), which constrains the reliability of PI estimation. Accurate PIs require a sufficiently large number of studies to provide a stable estimate of between-study variance (τ²); with small samples, prediction intervals often become excessively wide and unreliable, potentially leading to misinterpretation. Moreover, all included studies were observational cohorts with variations in patient populations, follow-up durations, and outcome definitions, further complicating the meaningful calculation of PIs. Future meta-analyses with a larger number of high-quality studies may benefit from including PIs to better contextualize between-study variability and generalizability of findings. Secondly, Many included studies were retrospective and non-randomized, introducing potential selection bias. Variability in follow-up duration, outcome definitions, and treatment protocols contributed to heterogeneity. In some studies, unbalanced group sizes may have disproportionately influenced pooled estimates. Moreover, differences in how outcomes like biochemical and clinical recurrence were defined limit direct comparability. Finally, publication bias and a lack of high-quality randomized controlled trials limit the generalizability of these findings.

Future research should focus on prospective, randomized trials comparing RP and BT, with standardized definitions for outcomes and long-term follow-up. Additionally, exploring genomic and molecular markers may help tailor treatment further. Novel imaging modalities like PSMA PET and risk prediction tools, including artificial intelligence algorithms or even canine olfactory detection, offer promising avenues for improving early diagnosis and personalized treatment planning. Evaluating patient-reported outcomes, including urinary, sexual, and bowel function, will also be critical for guiding shared decision-making and refining treatment guidelines. 

In conclusion, the results of this meta-analysis showed BT was significantly superior to RP in BCRFS outcomes among low- and intermediate-risk prostate cancer patients. The authors acknowledge that according to several guidelines on prostate cancer management, low-risk patients do not necessitate therapeutic intervention, so the authors performed a subgroup on risk stratification focusing on intermediate-risk prostate cancer patients only, which positive result revealed that BT remained superior to RP in intermediate-risk prostate cancer patients. The superiority of BT was also observed significantly in BCRFS and CRFS subgroups segmented by duration of follow-up. BT can be strongly considered as a therapeutic option for intermediate-risk prostate cancer patients. Nevertheless, the existing evidence coupled with our findings underlines the need for larger observational studies and longer follow-up to elucidate in more detail the superiority of both therapies on OS and CSS. Future studies should consider stratifying patients into high-risk patient who treated both therapies.

**Table 1:** Boolean Search Strategy

Boolean Operator	Syntax
AND	“Radical Prostatectomy” AND “Brachytherapy” AND “Prostate Cancer”
OR	"Brachytherapy" OR "BT"; “Radical Prostatectomy” OR “RP”
Combined	("Radical Prostatectomy" OR "RP") AND ("Brachytherapy" OR "BT") AND "Prostate Cancer"

**Table 2:** Study Characteristics

Authors, Year	Study Design	Country	Patients' Criteria of Eligibility	Total Participants (N)
Giberti, 2009 [21]	Observational cohort	Italy	* Patient with low-risk prostate cancer (clinical stage T1c or T2a, PSA value <10 ng/ml and Gleason sum <6) * In accordance with the ABS exclusion criteria included: previous pelvic irradiation, large median lobes, uroXow-Q max lower than 10 ml/s, history of multiple pelvic surgeries, previous transurethral resection of prostate, prostate volume greater than 60 ml and positive seminal vesicles biopsy	200
Ciezki, 2016 [20]	Cohort	USA	* Patients with NCCN-defined IRPC: PSA level 10-20 ng/ml, Gleason score ≤7, and stage cT1–T2c * Pathologic grading conformed to the Gleason grading from biopsy tissue	1818
Goy, 2019 [15]	Cohort	USA	* All patients were clinically staged, with a digital rectal exam for T-stage from the 2002 American Joint Committee Cancer staging * iPSA to treatment and biopsies of the prostate with Gleason score assessment * IRPC was classified as clinical stage T2b-c, Gleason score 3+4 (group 2) or 4+3 (group 3), and/or iPSA of 10.1-20.0 * PPBC>50% was calculated from the pathology report * Charlson co-morbidity index was assigned to each patient to assess overall health status	929
Zhou, 2019 [25]	Cohort	China	* All the patients performed according to the standards provided by the NCCN in IRPC and defined as: PSA 1020 ng/ml, or Gleason score 3+4=7, or tumor stage T2bT2c * Low risk is defined as: PSA <10 ng/ml and a Gleason score of ≤6 and tumor stage T1T2a * The inclusion criteria were the following: A clinical T-stage between T1c and T3a, ≥2 years follow-up post-treatment, and no distant metastasis * Patients who received adjuvant radiation therapy/chemotherapy and/or patients with distant metastasis were excluded from the present study	429
Hayashi, 2019 [22]	Cohort	Japan	* All patients had biopsy-proven prostate adenocarcinoma, and all external pathological specimens were reviewed by pathologists in our institution inhibitors, and alpha-blockers prior to the * The patients were categorized according to the US NCCN risk classification criteria, which defines “intermediate-risk” by 10 ≤ PSA level < 20 ng/ml, Gleason score ≤ 7, and stage cT1–T2c * Biochemical failure was defined for RP and BT by a nadir PSA level+2 ng/ml and for RP by a PSA level >0.2 ng/ml	1498
Tsumura, 2022 [24]	Cohort	Japan	* Candidates for the present study were patients with intermediate-risk prostate cancer who underwent SEED-BT plus or minus the combination of EBRT and RP at three tertiary hospitals between January 2006 and December 2011 * RP was performed via either the open retropubic approach or laparoscopic surgery * Patients with no evidence of BCR and < 4 years of follow-up were excluded	428
Liang, 2023 [23]	Cohort	China	* Patients were categorized according to the NCCN risk classification criteria, which defines IRPC by clinical stage T2b-c, Gleason score 3+4 (group 2) or 4+3 (group 3), and iPSA of 10.1–20.0 ng/ml * PPBC >50% was calculated from the pathology report * Favorable IRPC was described as patients with no more than one intermediate adverse risk factor, such as Gleason score 3+4 (group 2), iPSA 10.1-20.0 ng/ml, or clinical stage T2b-c, PPBC ≤50% * Those with multiple intermediate adverse risk factors, which included PPBC >50%, or any IRPC with Gleason score 4+3 (group 3), were classified as unfavorable IRPC	361

Note: iPSA = initial prostate-specific antigen; PPBC = percentage of positive biopsy cores; IRPC = intermediate-risk prostate cancer; ABS = American Brachytherapy Society; EBRT = external beam radiotherapy; NCCN = National Comprehensive Cancer Network; SEED-BT = seed brachytherapy; PSA = prostate-specific antigen; BCR = biochemical relapse.

**Table 3:** Characteristics of Patients Included in the Meta-Analysis by Study and Treatment Modality

Authors, Year	Initial PSA (ng/ml)		Maximum Follow-up (months)		Clinical T Stage (n)	Mean Age (years)	
	RP	BT	RP	BT		RP	BT
Goy, 2019 [15]	7.4 (Median)	8.2 (Median)	120 (Median)	110 (Median)	T1c (595) T2a (178) T2b (48) T2c (13) Unknown (95)	62:01:00	65:03:00
Ciezki, 2016 [20]	-	-	55.6 (Median)	48.9 (Median)	T1 or T2a (1237) T2b or T2c (544) T3 (42)	62	70
Giberti, 2009 [21]	7.8 (3.5) Mean (SD)	7.5 (2.9) Mean (SD)	60 (Median)	60 (Median)	T1c (123) T2a (77)	65:06:00	65:02:00
Hayashi, 2019 [22]	8.9 (10.5) Mean (SD)	7.6 (3.5) Mean (SD)	77	66	T1c (905) T2a-b (463) T2c (96) T3-4 (34)	66	70
Liang, 2023 [23]	12.0 (Median)	12.5 (Median)	54 (Median)	69 (Median)	T1c (57) T2a (76) T2b (73) T2c (155)	66	74
Tsumura, 2022 [24]	7.3 (Median)	7.9 (Median)	94 (Median)	96 (Median)	T1c (208) T2a-c (220)	68	69
Zhou, 2019 [25]	12.13 (6.00) Mean (SD)	13.25 (6.63) Mean (SD)	42.9 (Median)	50.1 (Median)	T1c (97) T2a (123) T2b (59) T2c (138) T3 (12)	65:28:00	73:04:00

Note: Initial PSA (ng/mL): Baseline prostate-specific antigen levels are shown as either median values or mean with standard deviation (SD), indicating the extent of variation in PSA levels between treatment groups. Maximum Follow-Up (months): The longest follow-up duration reported for each treatment group, expressed as median values, reflects the length of time over which outcomes were monitored. Clinical T Stage (n): The number of patients in each clinical T stage category (T1c through T3/T4), indicating tumor size and local extent, with grouping variations depending on the reporting style of the study. Mean Age (years): Average age of patients at the time of treatment for both RP and BT groups, offering insight into the age distribution and potential differences in patient selection between treatment modalities. Prostate-Specific Antigen (PSA); Standard Deviation (SD); radical prostatectomy (RP); brachytherapy (BT)

**Table 4:** Quality Assessment of Each Study by Newcastle Ottawa for Cohort Studies

Study	Selection^a^	Comparability^b^	Outcome^c^	Score	Interpretation
Goy, 2019 [15]	* *	*	* *	* * * * *	Fair
Ciezki, 2016 [20]	* * *	*	* *	* * * * * *	Good
Giberti, 2009 [21]	* * * *	*	* * *	* * * * * * * *	Good
Hayashi, 2019 [22]	* * * *	*	* *	* * * * * * *	Good
Liang, 2023 [23]	* * *	*	* *	* * * * * *	Good
Tsumura, 2022 [24]	* * *	*	* *	* * * * * *	Good
Zhou, 2019 [25]	* *	*	* *	* * * * *	Fair

^a^, Representativeness of intervention cohort, selection of non-intervention cohort, ascertainment of intervention, demonstration that outcome was not present at start of study. ^b^, Comparability of cohorts on basis of design or analysis. ^c^, Assessment of outcome, enough follow-up time length for outcome to occur, adequacy of follow-up cohorts

**Table 5:** Summary Oncological Outcome of Hazard Ratio (HR) in each Study

No	Author, Years	Treatment	Oncological Outcome (HR; (95% CI))			
			BCRFS	CRFS	OS	CSS
1	Goy, 2019 [15]	RP vs BT	0.61; (0.48-0.78)	1.14; (0.65-2.00)	1.20; (0.79-1.82)	1.08; (0.54-2.15)
2	Ciezki, 2016 [20]	Open & Laparoscopic RP vs LDR-BT	0.86; (0.78-0.94)	1.10; (0.83-1.47)	-	-
3	Giberti, 2009 [21]	RP vs BT	0.97; (0.37-2.57)	-	-	-
4	Hayashi, 2019 [22]	RP vs BT	0.81; (0.54-1.22)	-	0.82; (0.37-1.81)	-
5	Liang, 2023 [23]	RP vs LDR-BT	0.76; (0.63-0.91)	0.80; (0.31-2.07)	1.12; (0.80-1.57)	1.04; (0.79-1.38)
6	Tsumura, 2022 [24]	RP vs SEED-BT	0.69; (0.57-0.84)	1.22; (0.30-4.94)	0.97; (0.52-1.83)	1.15; (0.22-6.11)
7	Zhou, 2019 [25]	RP vs LDR-BT	1.17; (0.98-1.40)	0.79; (0.98-1.40)	-	-

## Author Contribution Statement

All authors contributed equally to the conception, design, data collection, analysis, and manuscript preparation.
